# Mechanistic Investigation of Exercise Interventions in Rodent Models of Alzheimer’s Disease and Prospects for Clinical Translation

**DOI:** 10.1155/np/6718671

**Published:** 2026-03-17

**Authors:** Tianhang Peng, Zike Zhang, Ni Ding, Jiayi Zhang

**Affiliations:** ^1^ School of Sports Science, Beijing Sport University, Beijing, 100084, China, bsu.edu.cn; ^2^ Hunan Normal University, Changsha, 410012, China, hunnu.edu.cn; ^3^ University of Science and Technology Beijing, Beijing, 100083, China, ustb.edu.cn

**Keywords:** β-amyloid (Aβ), Alzheimer’s disease, amyloid precursor protein (APP), exercise intervention, transgenic murine models

## Abstract

Alzheimer’s disease (AD) is a progressive and debilitating neurodegenerative disorder for which existing pharmacotherapies are inadequate to arrest pathological progression, highlighting the imperative to identify safe and effective nonpharmacological interventions. Exercise, as a multi‐target therapeutic modality, has been shown to reverse multiple facets of AD‐related neuropathology through diverse mechanisms. In this systematic review, we synthesize evidence on the effects of voluntary running, structured swimming, and modulation of the gut microbiota in transgenic murine models of AD. Exercise was found to ameliorate AD pathology by modulating amyloid precursor protein (APP) processing and β‐amyloid (Aβ) production/clearance, restoring mitochondrial integrity and function, attenuating neuroinflammatory responses, enhancing synaptic plasticity, and upregulating neurotrophic factors. Moreover, exercise reshapes the intestinal microbiome and thereby modulates the gut–brain axis, further promoting neuroimmune homeostasis and cognitive resilience. Through RNA sequencing data analysis, key genes such as Tlr4, Cdc42, and F13a1 were identified, which may play significant roles in neuroimmune regulation and cognitive protection. By integrating multi‐omics evidence, we propose a coordinated “exercise–microbiota–brain” mechanistic framework that offers theoretical support for personalized, exercise‐based therapeutic strategies and translational applications in AD. We also emphasize the necessity of future studies combining exercise with complementary interventions to accelerate the clinical translation of multimodal therapeutic approaches.

## 1. Introduction

Alzheimer’s disease (AD) is a prevalent late‐life disorder characterized by progressive cognitive decline and neurodegenerative pathology; its cardinal histopathological features include aberrant deposition of β‐amyloid (Aβ) plaques, neurofibrillary tangles (NFTs) formed by hyperphosphorylated tau, synaptic dysfunction, and a cascade of neuroinflammatory events. As the disease advances, patients progress from short‐term memory impairment and language deterioration to loss of independence in activities of daily living and, ultimately, death from multi‐system organ failure [1]. Global estimates from 2023 indicate that more than 55 million individuals are living with dementia attributable to AD [2], and the disorder imposes a markedly increased mortality risk in older adults (approximately a 40% elevation), together with substantial socioeconomic burden and rising health‐care expenditures, rendering AD a pressing public‐health challenge in aging societies [3].

Current therapeutic strategies for AD are broadly classified as pharmacological and non‐pharmacological. Pharmacotherapy—principally acetylcholinesterase inhibitors (e.g., donepezil, galantamine) and the NMDA receptor antagonist memantine—can transiently ameliorate cognitive deficits and behavioral and psychological symptoms of dementia (BPSD) but do not reverse underlying neuropathology [4, 5]. Multidomain nonpharmacological interventions (e.g., cognitive training and nutritional modulation) hold promise yet are constrained in clinical deployment by logistical complexity and implementation costs [5–12]. Exercise, as a nonpharmacological, multi‐target intervention, has garnered increasing interest in AD research; clinical studies indicate that regular physical activity not only lowers AD incidence risk but also improves cognitive outcomes by facilitating Aβ clearance, enhancing synaptic plasticity, and suppressing neuroinflammation, while avoiding many adverse effects and economic burdens associated with conventional pharmacotherapies [13–17].

The precise molecular mechanisms by which exercise confers neuroprotection remain incompletely defined, and transgenic murine models of AD provide indispensable platforms to interrogate these mechanisms. Owing to their short lifespan, controllable genetic background, and capacity to recapitulate hallmark AD pathologies (such as Aβ deposition and tauopathy), AD mouse models are central tools for mechanistic investigations [18, 19]. Widely used transgenic models differ substantially in pathological features, onset kinetics, and their suitability for specific lines of inquiry—factors that must be considered when interpreting exercise‐related effects. For example, amyloid precursor protein (APP)/PS1 mice harbor familial APP and PS1 mutations and display moderate‐rate Aβ plaque accumulation with progressive neuroinflammation but relatively weak tau pathology, making them well suited for studies of exercise effects on Aβ metabolism and inflammatory responses; the 5xFAD model carries five pathogenic mutations, exhibits rapid Aβ42 overproduction and abundant deposits by ~2 months of age with pronounced microglial activation and neuroinflammation, and is therefore appropriate for assessing interventions against rapidly progressing Aβ‐driven pathology; the 3xTg‐AD model manifests both Aβ and tau pathologies and thus better mirrors the multifaceted pathology of clinical AD, making it advantageous for studying exercise effects on synaptic plasticity, tau‐related signaling pathways, and interactions among coexisting pathologies [20, 21].

Recent studies have confirmed that exercise exerts neuroprotective effects through multiple pathways, including the activation of brain‐derived neurotrophic factor (BDNF) signaling, regulation of the autophagy‐lysosome pathway, and modulation of the gut‐brain axis [22–26]. However, the translational bottleneck across species and the dose–response relationship remain unclear. Recent review studies have discussed these issues, further strengthening the current research status and future directions of this field [27–33]. This study, based on a transgenic AD mouse model, systematically evaluates the effects of running, swimming, and gut microbiota modulation on AD mice, aiming to provide theoretical support for optimizing nonpharmacological treatments for AD and their clinical translation.

## 2. Methods

This study employed an integrative framework combining a systematic literature search with re‐analysis of publicly available transcriptomic datasets. Literature retrieval followed the PRISMA 2020 guidelines and applied the PICOS schema to define the scope: Population (P)—rodent models of AD; Intervention (I)—exercise‐related interventions (treadmill running, forced running, running wheel, swimming) and microbiota manipulations closely associated with exercise; Comparison (C)—non‐intervention or sedentary housing controls; Outcomes (O)—canonical AD pathological readouts; Study design (S)—preclinical animal experiments. Searches were performed in PubMed and Web of Science (databases indexed through November 2025) using keywords such as “Alzheimer’s disease,” “exercise,” “running,” “swimming,” “rodent models,” and “microbiota” (Table S1). The study selection workflow comprised: (i) title and abstract screening; (ii) full‐text assessment against PICOS criteria; (iii) exclusion of duplicate reports, studies lacking control groups, or manuscripts with insufficient description of models or interventions; (iv) independent screening by two reviewers with arbitration of discrepancies by a third reviewer; and (v) extraction of data stratified by exercise modality, model genotype, sex, intervention intensity, and primary outcomes (Figure S1). Methodological quality and risk of bias were appraised using the SYRCLE risk‐of‐bias tool for animal studies (Table S2) [34]. Two assessors independently scored each included study on the SYRCLE domains: studies adopting appropriate measures to mitigate bias were rated “low risk,” studies with insufficient information were rated “unclear risk,” and studies employing methods that likely increased bias were rated “high risk.” Items were scored numerically (“low risk” = 2, “unclear risk” = 1, “high risk” = 0) and summed to yield a composite quality score per study. Prior to assessment, the two evaluators underwent centralized training to harmonize scoring criteria; unresolved disagreements were referred to a third evaluator for adjudication.

For transcriptomic analyses, three publicly available RNA‐sequencing datasets were systematically re‐analyzed: (1) GSE185407—male APPPS1‐21 mice, 4 months old, dorsal cortex, antibiotic (ABX) or control treatment, *n* = 77, sequenced on Illumina HiSeq 4000 and NovaSeq 6000; (2) GSE154428—male 5xFAD mice at 4 and 10 months, hippocampus, ABX or control, *n* = 28, sequenced on Illumina HiSeq 1000; and (3) GSE164618—male and female AD mice subjected to 4‐month methionine‐restriction (MR) intervention, hippocampus, *n* = 85, sequenced on BGISEQ‐500. Expression matrices were normalized according to input data type: raw count matrices (GSE185407 and GSE164618) were processed with DESeq2 (v1.38.0) using the median‐of‐ratios method for normalization and dispersion estimation, and log2 fold‐change estimates were stabilized using lfcShrink (type = “ashr”); matrices provided as logCPM or FPKM/TPM (GSE154428) were analyzed with limma (v3.52.2) in conjunction with voom (quality weights) to fit weighted linear models. Batch effects were corrected with limma::removeBatchEffect(). Differentially expressed genes (DEGs) were identified using a unified threshold of |log2FC| > 1 and FDR < 0.05 (Benjamini–Hochberg correction); cross‐cohort integration analyses applied an additional BH correction to bolster statistical robustness. Significantly dysregulated genes were subjected to Gene Ontology (GO) and Kyoto Encyclopedia of Genes and Genomes (KEGG) enrichment analyses via clusterProfiler (v4.4.4), with visualization implemented using enrichplot and ggplot2; enrichment terms were considered significant only if *p* < 0.05 and *q* < 0.05. Intersections of DEGs across the three cohorts were computed with the VennDiagram package.

To interrogate shared molecular networks across intervention conditions, the intersecting DEG set (*n* = 9151) was uploaded to the STRING database (version 11.5) to construct a protein–protein interaction (PPI) network under a stringent high‐confidence threshold (combined score > 0.95). Given the very large gene set, lower confidence cutoffs (e.g., 0.7) risked incorporation of numerous text‐mining or prediction‐derived, lower‐reliability edges, precipitating exponential network expansion, degree distribution homogenization, and distortion of local and global topological features—outcomes that erode biological interpretability. Therefore, a conservative confidence threshold was adopted to maximize interaction reliability. The resulting high‐confidence PPI network was visualized and reconstructed in Cytoscape (v3.6.1). Network topology metrics, including degree centrality, betweenness centrality, and closeness centrality, were computed using the NetworkAnalyzer module. In pre‐analyses we observed high concordance among centrality rankings in the high‐threshold network, which limited discriminatory utility of multi‐metric aggregation; consequently, for methodological parsimony and result robustness we selected node degree as the principal criterion to identify hub genes and extracted the top 20 nodes by degree as candidate core regulators putatively mediating shared responses to ABX and MR interventions. This strategy yielded a structurally stable, low‐noise set of key regulatory genes to guide subsequent mechanistic dissection of gut–brain axis interventions in AD models.

## 3. Neuroprotective Mechanisms of Running Exercise in AD

Running paradigms (including treadmill and running‐wheel modalities) are the most commonly employed exercise interventions in AD mouse models (Table 1). Preclinical studies in rodent models indicate that both voluntary and forced running, as nonpharmacological interventions, markedly ameliorate AD‐related pathology via pleiotropic mechanisms. This section synthesizes current evidence on how exercise influences Aβ homeostasis, neuroimmune regulation, mitochondrial quality control, and neuronal plasticity.

**Table 1:** Effects of running exercise on a murine model of Alzheimer’s disease.

Experimental model	Sample groups	Exercise intervention	Intensity	Duration	Frequency	Main findings	Underlying mechanisms	Cognitive and behavioral test results
Tg2576 mouse [35]	AD (*n* = 10) <br > AD + Low Ex (*n* = 10) <br > AD + High Ex (*n* = 10)	Treadmill training	Low: 15 m/min; <br > High: 32 m/min; <br > Incline: 10%	12 weeks	60 min/day, 5 days/week	After exercise: decreased cortical Aβ40/42 levels; increased HSP70 expression	Exercise upregulates Aβ‐clearance proteins in the brain, dose‐dependently reducing Aβ deposition	Not assessed
NSE/APP transgenic mouse [36]	CON (*n* = 8) <br > AD (*n* = 8) <br > AD + Exercise (*n* = 8)	Progressive running	Weeks 1–4:10 m/min; <br > Weeks 5–12:12 m/min	12 weeks	30 min/day → 60 min/day, 5 days/week	Increased SIRT1 activity; decreased Aβ42; increased RARβ, ADAM10; decreased ROCK1	Exercise regulates the non‐amyloidogenic pathway via SIRT1, inhibits BACE1 and inflammation, improving Aβ metabolism	MWM shows significant cognitive improvement after treadmill exercise
APP/PS1 mouse [37]	CON (*n* = 6) <br > Exercise only (*n* = 6) <br > AD (*n* = 6) <br > AD + Exercise (*n* = 6)	Treadmill training	5 m/min for 5 min; <br > 8 m/min for 5 min; <br > 11 m/min for 20 min	5 months	30 min/day, 6 days/week	Decreased hippocampal and cortical Aβ plaques; increased neuron density; increased GFAP and APP; decreased BACE1 expression	Inhibits Aβ production and transport, enhances neuroprotective mechanisms	Not assessed
3xTg‐AD mouse [38]	AD (*n* = 10) <br > AD + Exercise (*n* = 10) <br > Control (*n* = 10)	Progressive running	6 m/min, 5 min; <br > 9 m/min, 5 min; <br > 12 m/min, 20 min; <br > 15 m/min, 5 min; <br > 12 m/min, 5 min	12 weeks	40 min/day, 5 days/week	Increased mitochondrial function; increased NRF1 and PGC‐1 α expression	Activates mitochondrial biogenesis and antioxidant signaling pathways, enhances neurotrophic factor expression	MWM shows cognition in the exercise group restored to normal
AD model mouse [39]	AD + Exercise (*n* = 8) <br > AD (*n* = 8)	Treadmill training	15 m/min	7 days	30 min/day, daily	Decreased TNF‐α and IL‐1β; increased neurogenesis; decreased p38 and JNK phosphorylation	Exercise modulates MAPK signaling, improves brain immune status and neurogenesis	MWM shows improved cognition in the running group
3xTg‐AD mouse [40]	AD (*n* = 9) <br > AD + 1 d/week Ex (*n* = 8) <br > AD + 3 d/week Ex (*n* = 8) <br > Control (*n* = 8)	Treadmill training	8 m/min	12 weeks	60 min/day, 1 or 3 days/week	Decreased serum and brain MCP‐1; increased RANTES levels	Exercise modulates inflammation‐related chemokine expression, alleviating neuroinflammation	Not assessed
Tg2576 mouse [41]	AD + voluntary Ex (*n* = 10) <br > AD + forced Ex (*n* = 10) <br > AD + forced Ex control (*n* = 9) <br > AD (*n* = 9)	Running + voluntary activity	VOL: voluntary intensity; <br > FOR: forced (same as VOL); <br > FCON: forced control (same as VOL)	16 weeks	1 h/day, 5 days/week	Decreased Aβ plaques; increased hippocampal volume	Exercise promotes neuronal plasticity, maintaining hippocampal function	NOR shows highest cognitive scores in the voluntary exercise group
APP/PS1 mouse [42]	AD + Exercise (*n* = 10) <br > AD (*n* = 10) <br > Control (*n* = 10)	Progressive running	10 m/min	4 months	20 min/day, 5 days/week	Increased hippocampal, CA1/2, CA3 and DG volumes and neuron count; improved dendritic complexity	Exercise activates BDNF‐AKT/PKC/ERK signaling, enhances LEP1 expression, improving neural plasticity	MWM and visible‐platform tests show improved cognition in the running group
APP/PS1 mouse [43]	AD + Exercise (not reported) <br > AD (not reported)	Progressive running	10 → 12 m/min	9 weeks	20 → 60 min/day, 5 days/week	Restored memory; increased CA1/CA3 neuron dendritic complexity; decreased Aβ levels	Exercise enhances BDNF signaling, protecting amygdala and hippocampal neurons from AD‐related damage	STM shows improved cognition in the running group

*Note*: This table summarizes experimental paradigms, cognitive outcomes, and putative molecular mechanisms of running interventions in animal models of AD. The table catalogs intervention modalities (voluntary wheel running, voluntary wheel access, or treadmill training), intervention duration and frequency, and cognitive assays employed (e.g., Morris water maze [MWM], Y, maze [YM], novel object recognition [NOR]). Running regimens typically produce robust improvements in learning and memory in AD models that are accompanied by reductions in cerebral β‐amyloid (Aβ) burden and tau phosphorylation, attenuation of neuroinflammatory mediators (e.g., interleukin‐1β [IL‐1β], tumor necrosis factor‐α [TNF‐α]), and upregulation of neurotrophic factors such as brain‐derived neurotrophic factor (BDNF), glial cell line‐derived neurotrophic factor (GDNF), and nerve growth factor (NGF).

Abbreviations: AD, Alzheimer’s disease; Aβ, β‐amyloid; BDNF, brain‐derived neurotrophic factor; GDNF, glial cell line‐derived neurotrophic factor; IL‐1β, interleukin‐1β; LTP, long‐term potentiation; MWM, Morris water maze; NGF, nerve growth factor; NOR, novel object recognition; TNF‐α, tumor necrosis factor‐α; YM, Y‐maze.

### 3.1. Aβ Homeostasis and Metabolic Reprograming

Running exerts bidirectional regulation of Aβ production and clearance, thereby preserving Aβ dynamic equilibrium. In Tg2576 mice, 12 weeks of treadmill training robustly upregulated proteolytic enzymes such as neprilysin (NEP), insulin‐degrading enzyme (IDE), and MMP9, as well as Aβ efflux transporters including LRP1 and HSP70, producing a dose‐dependent reduction in soluble cortical and hippocampal Aβ40/42 levels [35]. This clearance mechanism is tightly linked to exercise‐induced metabolic reprograming: treadmill exercise increases cerebral SIRT1 expression, which, via downregulation of ROCK1 and upregulation of RARβ, promotes ADAM10 expression while suppressing BACE1, biasing APP processing toward the non‐amyloidogenic pathway and ultimately reducing Aβ generation and deposition [36]. Long‐term interventions corroborate the translational potential of this axis: 5 months of treadmill training in APP/PS1 mice lowered BACE1 and presenilin‐1 (PS1) levels, inhibited amyloidogenic processing, and downregulated RAGE expression to reduce Aβ re‐entry into the brain [37]. Importantly, the temporal window of exercise critically influences outcome—early‐stage 12‐week treadmill intervention in 3xTg‐AD mice enhanced selective mitophagy of damaged mitochondria and thereby prevented Aβ‐associated mitochondrial dysfunction [38]. Moreover, PPAR agonists have shown promise in exercise‐mediated blood–brain barrier (BBB) modulation, improving barrier integrity and facilitating Aβ efflux [44].

### 3.2. Neuroimmune Modulation and Inflammation Attenuation

Exercise counteracts AD‐associated neuroinflammation by modulating cytokine profiles and remodeling glial phenotypes. In Aβ‐rich environments, treadmill running suppresses overactivation of MAPK cascades (ERK, p38, JNK), significantly lowering pro‐inflammatory cytokines such as TNF‐α and IL‐1β and attenuating astrocytic reactivity [39]. A dose‐dependent anti‐inflammatory effect of exercise was demonstrated in 3xTg‐AD mice: 12 weeks of running‐wheel training restored serum and brain chemokine levels (e.g., RANTES, MCP‐1) to baseline only under high‐frequency regimens (three sessions per week), indicating that adequate exercise intensity is required for meaningful inflammation resolution [40]. Mechanistically, voluntary running often outperforms forced exercise in reducing plaque burden and ameliorating memory deficits, a discrepancy that may be attributable to differential stress‐hormone responses—Tg2576 mice with access to voluntary wheels exhibited fewer Thioflavin‐S‐positive plaques and superior novel object recognition relative to treadmill‐trained counterparts [41].

### 3.3. Mitochondrial Repair and Mitigation of Oxidative Stress

Running enhances mitochondrial quality control and thereby improves neuronal viability in AD. In early‐stage 3xTg‐AD mice, 12 weeks of treadmill exercise restored the balance between mitochondrial fusion and fission, promoted selective sequestration of damaged mitochondria followed by lysosomal degradation, preserved a healthy mitochondrial pool, reinstated energy metabolism, and stimulated neurogenesis [38]. This mitochondrial restorative capacity is intimately linked to reduction of oxidative stress: prior studies have shown that exercise reduces markers of oxidative damage in the brains of TgCRND8 mice and reverses autophagic dysfunction [45, 46].

### 3.4. Structural and Functional Neuroplasticity

Exercise promotes synaptic repair and neurogenesis via the BDNF–TrkB signaling axis. In APP/PS1 mice, 4 months of running increased hippocampal volume and neuronal density in the CA1/CA3/dentate gyrus (DG) subfields, concomitantly ameliorating spatial memory deficits in the Morris water maze [42]. This structural remodeling extends to limbic circuitry: 10 weeks of treadmill training in transgenic mice augmented dendritic arborization of basolateral neurons and elevated phosphorylated TrkB, p‐AKT, and p‐PKC levels, thereby rescuing amygdala‐dependent memory through BDNF‐dependent synaptic plasticity mechanisms [43]. Voluntary wheel running further potentiated these effects: in aged SAMP8 mice, 6 months of wheel access reversed hippocampal aging phenotypes, including expansion of the vascular network, upregulation of COL1A1/COL1A2, and suppression of extracellular‐matrix degradation markers (FMOD, PTGDS) [47]. Exercise also exerts robust modulation of synaptic plasticity, notably long‐term potentiation (LTP) and long‐term depression (Ltd). Voluntary wheel running enhances LTP in the hippocampal CA1 region and improves NMDA receptor–mediated synaptic transmission—mechanisms central to learning and memory consolidation [48]. In APP/PS1 and other AD models, voluntary exercise partially restores Aβ‐induced LTP impairments and attenuates aberrantly potentiated Ltd, thereby stabilizing synaptic homeostasis and network function [49, 50]. Moreover, exercise upregulates synaptic proteins and amplifies BDNF–TrkB signaling, collectively strengthening synaptic architecture and plastic potential [51–53]. These findings indicate that reversal of synaptic‐plasticity deficits is a principal mechanism by which exercise improves cognition in AD models.

### 3.5. Temporal Dynamics and Exercise Parameters

The temporal window and modality of exercise critically influence therapeutic outcomes. Early interventions maximize neuroprotective benefits—for example, a 12‐week treadmill regimen initiated prior to symptom onset in 3xTg‐AD mice prevented cognitive decline by restoring mitochondrial function [38]. Sustained treadmill exercise can substantially reduce Aβ burden in APP/PS1 mice, decreasing both plaque number and area [37]; nevertheless, late‐stage exercise may still confer partial benefits, such as reduced Aβ load and microgliosis in TgCRND8 mice [54]. Voluntary running tends to outperform forced exercise in augmenting hippocampal volume and reducing insoluble Aβ aggregation, a discrepancy plausibly attributable to differences in stress‐axis activation [41]. Importantly, exercise frequency exhibits a threshold effect: anti‐inflammatory chemokine normalization required a minimum regimen (e.g., ≥3 sessions per week) to achieve significant reduction in inflammatory mediators [40].

### 3.6. Conclusions and Translational Outlook

Preclinical rodent studies consistently demonstrate that running mitigates AD pathology through multifaceted mechanisms—including modulation of the Aβ cascade, attenuation of neuroinflammation, restoration of mitochondrial function, and enhancement of synaptic plasticity. Mechanistic nodes such as BDNF–TrkB–driven neurogenesis, SIRT1‐mediated metabolic reprograming, and LRP1‐dependent Aβ clearance provide coherent theoretical frameworks for clinical translation. Remaining challenges include delineating optimal exercise “doses” across AD stages (intensity, duration, modality), clarifying peripheral–central immune crosstalk, and characterizing sex‐specific responses to exercise. Future investigations that integrate multi‐omics profiling will be instrumental in mapping exercise‐induced epigenetic and proteostatic networks and in designing personalized, evidence‐based exercise prescriptions for AD prevention and adjunctive therapy.

Notably, there is heterogeneity in MWM and NOR probe‐trial designs and in the control of motor/sensory confounds across studies. Inter‐session intervals (ISI) for MWM probe trials vary (e.g., Kim et al. [38] used a 15 min ISI), whereas Sun et al. [39] and Koo et al. [36] implemented staged testing but did not clearly report ISI. NOR probe intervals span short‐ to intermediate‐term memory windows (e.g., 10 min in Sun et al. [39] vs., 50 min in Yuede et al. [41]). Regarding motor and sensory controls, Koo et al. [36] and Sun et al. [39] conducted visible‐platform trials after MWM to exclude confounding effects of swim ability or visual deficits, whereas Yuede et al. [41] and Kim et al. [38] only reported no significant intergroup differences in basic locomotor capacity—providing limited but necessary evidence of motor control. Many other studies did not directly assess motor or sensory function, indicating that cognitive outcomes should be interpreted cautiously given potential confounding by differences in physical ability. Finally, the preponderance of included studies used forced‐treadmill paradigms and did not measure circulating stress markers such as plasma corticosterone (CORT) or corticotropin‐releasing hormone (CRH), with the exception of Yuede et al. [41]; therefore, the reported benefits of exercise may be partially confounded by stress responses.

## 4. Molecular Mechanisms of Swimming Exercise in AD: From Redox Homeostasis to Neuroimmune Remodeling

Swimming, as a low–mechanical‐stress, whole‐body aerobic modality, elicits multidimensional neuroprotective effects in rodent models of AD (see Table 2). Distinct from terrestrial running, the unique hydrodynamic characteristics of the aquatic environment (buoyancy, resistance) not only reduce joint loading but also provoke systemic metabolic reprograming and modulation of BBB function, thereby targeting core AD pathologies—Aβ deposition, tau hyperphosphorylation, oxidative stress, and neuroinflammation. This section integrates molecular, cellular, and behavioral evidence to delineate the cascade of regulatory networks engaged by swimming interventions.

**Table 2:** Effects of swimming exercise on a murine model of Alzheimer’s disease.

Experimental model	Sample groups	Exercise intervention	Intensity	Duration	Frequency	Main findings	Underlying mechanisms	Cognitive and behavioral test results
STZ rat model [55]	CON (*n* = 8–10) <br > Ex (*n* = 8–10) <br > AD (*n* = 8–10) <br > AD + Ex (*n* = 8–10)	Swimming	Voluntary intensity	26 days	10 min/day → 1 h/day, 5 days/week	Increased synaptic proteins; decreased inflammatory markers and oxidative stress levels	Exercise activates Nrf2/ARE pathway, enhances antioxidative capacity, inhibits glial activation and synaptic damage	BMT, NOR show improved cognition in the swimming group
AD mouse model [56]	CON (*n* = 6) <br > AD (*n* = 6) <br > AD + voluntary exercise (*n* = 6) <br > AD + forced exercise (*n* = 8) <br > AD + forced exercise control (*n* = 6)	Swimming	Voluntary group: voluntary intensity; <br > Loaded group: 5% body weight	90 days	20 min/day → 1 h/day, daily	Increased NGF and BDNF; decreased MDA and protein carbonyl; increased GSH and SOD activity	Exercise alleviates oxidative stress, enhances neurotrophic support, improves cognitive and behavioral performance	OFP, EPM, MWM show improved cognition in the swimming group
AD mouse model [57]	CON (*n* = 10) <br > AD (*n* = 10) <br > Exercise (*n* = 10) <br > AD + Exercise (*n* = 10)	Loaded swimming	Load 0%–3% body weight	8 weeks	60 min/day, 5 days/week	Increased reactive species (RS), TNF‐α, IL‐1β; increased SOD, GPx, GST activities	Swimming enhances the antioxidant system, suppresses inflammatory factor expression, protects frontal and hippocampal function	OFT, STM, LTM show improved cognition in the swimming group
3xTg‐AD mouse [58]	AD (*n* = 4) <br > AD + Exercise (*n* = 4)	Swimming	Voluntary intensity	4 weeks	30 min/day, 5 days/week	Upregulation of 412 pathways related to antiviral and interferon response; downregulation of pathways related to axon development and metal ion homeostasis	Exercise modulates neuroinflammation via interferon signaling, and affects Aβ–neuron interactions via ECM downregulation	Not assessed
AD mouse model [59]	CON (not reported) <br > AD (not reported) <br > AD + Vit D (not reported) <br > AD + Exercise (not reported) <br > AD + Vit D + Exercise (not reported)	Free swimming	Voluntary intensity	4 weeks	30 min/day, daily	After combined exercise and vitamin D: increased GSH and IL‐10; decreased IL‐6 and LDA; increased neurotransmitter levels	Exercise activates PI3K/AKT pathway, inhibits GSK‐3β activity, enhances cholinergic function and dopamine signaling, alleviates neuroinflammation	MWM shows swimming improved cognition; combined exercise and vitamin D yielded even greater improvement
AD mouse model [60]	CON (*n* = 8) <br > AD (*n* = 8) <br > Exercise (*n* = 8) <br > AD + Exercise (*n* = 8)	Progressive loaded swimming	Tail load 0%–3% body weight	8 weeks	60 min/day, 5 days/week	Increased BDNF, GDNF, CDNF, NGF; decreased TRP, KYN, NF‐κB and other inflammatory markers	Exercise regulates the TRP–KYN metabolic pathway, reduces IDO activity, improves neurotrophic factor expression, combining anti‐inflammatory and neuroprotective effects	NOR, TST, EPM show improved cognition in the swimming group

*Note*: This table summarizes experimental designs, cognitive outcomes and putative molecular mechanisms of swimming interventions in animal models of AD. It catalogs various swimming paradigms (e.g., intermittent vs., continuous protocols, duration and frequency) and associated cognitive assessments (for example, the Morris water maze). Studies report that swimming improves cognitive deficits in AD models—manifested as enhanced spatial learning and memory—accompanied by reductions in cerebral Aβ plaque burden, suppression of neuronal apoptosis, enhancement of mitochondrial biogenesis, upregulation of neurotrophic factors such as brain‐derived neurotrophic factor (BDNF) and glial cell line‐derived neurotrophic factor (GDNF), and attenuation of neuroinflammatory responses.

Abbreviations: AD, Alzheimer’s disease; Aβ, β‐amyloid; BDNF, brain‐derived neurotrophic factor; GDNF, glial cell line‐derived neurotrophic factor; IL‐1β, interleukin‐1β; LTP, long‐term potentiation; MWM, Morris water maze; NGF, nerve growth factor; NOR, novel object recognition; TNF‐α, tumor necrosis factor‐α; YM, Y‐maze.

### 4.1. Bidirectional Regulation of Aβ Metabolism and Tau Pathology

Swimming promotes Aβ clearance while suppressing amyloidogenic processing, thus reconfiguring Aβ dynamics. In streptozotocin (STZ)‐induced sporadic AD rats, swimming preconditioning significantly reduced hippocampal Aβ(1–42) levels and attenuated tau phosphorylation at Ser396 and Thr231 residues [55]. This dual action is plausibly mediated by activation of the Nrf2/ARE axis: swimming upregulated heme oxygenase‐1 (HO‐1) and NAD(P)H:quinone oxidoreductase 1 (NQO1), enhancing antioxidant defenses, limiting Aβ‐induced oxidative neuronal injury, and indirectly restraining aberrant tau phosphorylation [55]. Long‐term swimming (90 days) has been shown to modulate APP‐processing enzyme activities by downregulating β‐secretase (BACE1) while upregulating the α‐secretase ADAM10, thereby diverting APP toward the non‐amyloidogenic pathway and reducing de novo Aβ production [56]. Notably, swimming‐mediated Aβ clearance may exhibit spatiotemporal specificity: in an intracerebroventricular Aβ1‐40 injection model, 8 weeks of swimming enhanced activity of the BBB efflux transporter LRP1 and substantially lowered Aβ burden in the hippocampus and prefrontal cortex [57]. These observations suggest modality‐dependent preferences in Aβ clearance pathways.

### 4.2. Redox Homeostasis and Mitochondrial Repair

Swimming activates intrinsic antioxidant systems and significantly reverses AD‐associated oxidative stress. Transcriptomic profiling revealed that swimming upregulated components of the thioredoxin system (e.g., Txnrd1, Txnip) and the rate‐limiting enzyme for glutathione synthesis (Gclc) in the cortex of 3xTg‐AD mice, concomitant with suppression of NADPH oxidase (NOX2) activity, thereby reducing reactive oxygen species (ROS) generation at its source [58]. Furthermore, swimming promotes mitochondrial biogenesis via PGC‐1α and facilitates Parkin‐dependent mitophagy, rescuing Aβ‐induced collapse of mitochondrial membrane potential and defects in ATP synthesis [57]. Mitochondrial quality control was further potentiated when swimming was combined with vitamin D supplementation, manifested by increased complex I activity and reduced cytochrome‐c release, implying synergistic interactions between exercise and nutritional modulators [59].

### 4.3. Attenuation of Neuroinflammation and Remodeling of the Immune Microenvironment

Swimming remodels the glia–neuron interactome to sculpt a less pro‐inflammatory neural milieu. In the STZ model, swimming preconditioning reversed STZ‐induced reductions in presynaptic and postsynaptic markers (synaptophysin and PSD‐95) in hippocampal CA1, and, through activation of the Nrf2/ARE pathway, augmented antioxidant enzyme expression—consequently suppressing glial proliferation and release of proinflammatory cytokines and providing robust neuroprotection [55]. Single‐cell transcriptomic analyses further demonstrated that swimming selectively downregulated cortical gene modules associated with chemokine signaling and learning/memory deficits while upregulating anti‐inflammatory regulatory factors, indicating precise reprograming of neuroimmune networks [58]. Of particular note, swimming exerts unique effects on tryptophan metabolism: in an Aβ1‐42 model, swimming normalized prefrontal cortical levels of kynurenine (KYN) to control values and inhibited indoleamine 2,3‐dioxygenase (IDO) activity, thereby preventing accumulation of the neurotoxic metabolite quinolinic acid and ameliorating depressive‐like behaviors [60].

### 4.4. Comparison of Exercise Modalities and Translational Implications

Compared with terrestrial running, swimming exhibits distinctive mechanistic advantages and specific clinical applicability in AD interventions: (1) with regard to antioxidant defenses, swimming more effectively counters oxidative insult through activation of the Nrf2/ARE pathway and synergistic interactions with vitamin D supplementation [59]; (2) at the level of immune modulation, swimming selectively suppresses the IDO–KYN axis, rendering it particularly effective for ameliorating mood‐related cognitive disturbances [60]; and (3) owing to its low joint‐loading properties, swimming is especially well suited for AD patients with comorbid degenerative joint disease [61]. However, swimming appears comparatively less potent in driving Aβ efflux and Parkin‐dependent mitophagy, suggesting that hybrid or combined exercise regimens may yield complementary benefits by engaging both clearance and mitochondrial quality‐control pathways [41, 57]. Future studies should integrate multimodal neuroimaging and liquid‐biopsy modalities to quantify the dynamic effects of swimming on AD biomarkers (e.g., plasma p‐tau181, neurofilament light chain) and to delineate dose–response relationships between swimming exposure and cognitive preservation, thereby providing empirical foundations for personalized exercise prescriptions in clinical translation.

The MWM is used to evaluate spatial learning and long‐term memory; Belviranlı et al. [56] employed a 24 h probe to assess memory retention. The NOR task probes recognition memory—Souza et al. [57] and Souza et al. [60] used 90 min and 24 h probe intervals respectively to capture short‐ and long‐term memory, whereas Wu et al. [55] uniformly used a 24 h delay. The Barnes maze also assesses spatial memory with a 24 h probe interval in Wu et al. [55]. Medhat et al. [59] employed the spontaneous alternation Y‐maze to index working memory; as a single‐session paradigm it does not include an independent probe phase. Regarding control for motor confounds, Souza et al. [57], Souza et al. [60], and Belviranlı et al. [56] evaluated locomotor activity (e.g., open‐field) to exclude performance bias, whereas Medhat et al. [59] and Wu et al. [55] did not report motor assessments and thus their cognitive outcomes should be interpreted with caution. Additionally, Widjaya et al. [58] did not include behavioral testing or motor‐function evaluation.

## 5. The Gut–Brain Axis and Exercise Intervention: A Novel Microecological Avenue for AD Therapy

### 5.1. Molecular Dissection of Targeted Microbiota Interventions in AD Models

The gut microbiota—by virtue of its vast gene repertoire (~150‐fold that of the human genome) and its profound regulation of host immune, metabolic, and neural functions—has been dubbed the “second brain” [62–64]. The microbiota–gut–brain axis exerts bidirectional control over the central nervous system via neural, immune, and metabolic pathways, substantially influencing CNS function and homeostasis. Clinical studies have documented characteristic dysbiosis in the gut microbiota of AD patients, manifested as reduced α/β diversity, an increased relative abundance of Bacteroidetes, and a marked depletion of Firmicutes and neuroprotective taxa such as Bifidobacterium [65]. A significant correlation was observed between the clinical severity scores of AD patients and the abundance of altered microbiomes in a Chinese cohort [66]. Cattaneo and colleagues further demonstrated upregulation of pro‐inflammatory taxa (e.g., Escherichia–Shigella) and downregulation of anti‐inflammatory taxa (e.g., Ruminococcus), changes that were strongly associated with elevated plasma inflammatory markers such as IL‐6 and TNF‐α [67]. In APP/PS1 transgenic mice, dysbiosis emerges as early as 3 months of age and is associated with exacerbated cerebral Aβ deposition by 6 months, suggesting that gut microbiota alterations may act as an early pathological trigger in AD [68, 69]. This notion aligns with the “microbiota–amyloid cascade hypothesis” proposed by Harach et al., whereby microbial metabolites compromise the BBB, precipitate neuroinflammation, and thereby promote Aβ aggregation and neurodegeneration [70]. Accordingly, interventions targeting the gut microbiota have emerged as a promising avenue in AD therapeutics. Preclinical studies indicate that ABX–induced microbiota perturbation can attenuate cerebral Aβ accumulation and alter microglial activation in male mice [71–73]. Moreover, dietary methionine restriction (MR)—a nutritional intervention—has been shown to ameliorate cognitive deficits in AD mouse models via the gut–brain axis, with putative mechanisms involving modulation of methylation metabolism that links microbial activity to cerebral metabolic signaling [74–76].

The microbiota shifts induced by MR reflect specific metabolic drivers rather than a nonspecific nutrient‐deficiency effect. Methionine, as the central substrate for sulfur‐amino‐acid metabolism, S‐adenosylmethionine (SAM) synthesis, glutathione (GSH) production, and polyamine metabolism, reshapes host–microbial co‐regulated metabolic networks when reduced [77]. MR decreases the luminal supply of sulfur substrates, thereby suppressing sulfide‐dependent taxa (e.g., Desulfovibrio, Bilophila) while selectively promoting expansion of microbes capable of catabolizing single‐carbon or alternative carbon sources; concurrently, MR lowers host oxidative stress and shifts the microbiota toward taxa with greater antioxidant capacity [77, 78]. Thus, MR‐driven microbiota remodeling displays pronounced metabolic specificity. It is important to note that methionine is not the only dietary amino acid capable of substantially altering microbial ecology: distinct amino acids can directionally reconfigure the microbiota via their specific metabolic pathways. For example, tryptophan restriction reduces indole‐producing taxa and weakens microbiota‐mediated gut–brain signaling; branched‐chain amino acid (BCAA) restriction increases *Akkermansia* spp., which is associated with metabolic improvements; and arginine restriction modulates gut immunity by affecting NO‐related symbionts [79–83]. Collectively, these studies indicate that targeted amino acid manipulations can elicit predictable, host–microbe metabolic axis–mediated shifts in community composition.

To further explore the molecular mechanisms of microbiota intervention in AD, this study integrated RNA‐seq data from AD mice in the GEO database and compared gene expression differences between the ABX and MR intervention groups and the control group. Heatmaps (Figure 1A–C) display the top 20 DEGs in the intervention and control groups, which are involved in multiple key AD mechanisms, including neurofunction, inflammation regulation, and synaptic transmission. Abnormal expression of Hspa1a may be associated with exacerbated neuronal damage; overexpression of App may promote the deposition of Aβ; dysregulation of Tnfaip6 may be related to the acceleration of neurodegeneration; and dysregulation of Cdh2 may affect synaptic adhesion and cognitive function. These significantly DEGs provide potential research targets for further investigation of the molecular mechanisms underlying AD. These prominent DEGs provide candidate molecular targets for mechanistic dissection of AD pathogenesis. GO and KEGG enrichment analyses performed on three transcriptomic cohorts (GSE185407, GSE154428, GSE164618) revealed that microbiota‐targeted interventions (ABX treatment and MR diet) significantly modulate multiple key biological pathways in AD model mice. In APPPS1‐21 mice following ABX treatment, GO analysis highlighted upregulation of terms such as “sperm flagellar motility” and “chemokine activity,” suggestive of altered immune cell chemotaxis and reproductive‐related gene expression; KEGG enrichment implicated pathways including “parathyroid hormone synthesis and action,” “rheumatoid arthritis” and “prolactin signaling,” collectively pointing to systemic effects on calcium–phosphate metabolism, endocrine regulation, and inflammatory responses (Figure 2A). Analysis of 5xFAD mice yielded comparable trends: GO terms such as “positive regulation of interleukin‐1β production” and “phosphatidylethanolamine binding” implicate inflammatory signaling and lipid metabolism; KEGG hits including “fat digestion and absorption,” “glutathione metabolism,” “asthma,” and “HIV‐related pathways” further support the notion that ABX treatment may influence AD progression via multiple metabolic and immune axes (Figure 2B). By contrast, MR diet interventions were predominantly enriched for pathways related to synaptic function and lipid metabolism—for example, “synaptic vesicle cytoplasm” and “unsaturated fatty acid biosynthesis”—suggesting neuroprotection via enhancement of neuronal function and bioenergetic homeostasis (Figure 2C). Cross‐comparison of DEGs across all three datasets identified 9151 shared genes (Figure 2D), reflecting a conserved molecular response to distinct microbiota‐targeted manipulations in AD models. The high‐confidence PPI network constructed based on these co‐expressed genes (Figure 2E,F) reveals several protein nodes that may play central regulatory roles in AD. Although genes such as Tlr4, Cdc42, and F13a1 exhibit high centrality within the network, it is important to note that the centrality of a network node does not directly indicate its causal role. For example, Tlr4 has already been demonstrated through functional experiments to play an important role in microglial inflammation activation, Aβ‐induced neurotoxicity, and immune regulation, and thus can be considered a potential key gene supported by experimental evidence [84]. However, genes such as Ms4a1, Cdc42, and F13a1, while showing high connectivity in the network, currently lack sufficient direct experimental data to confirm their causal role in the pathogenesis of AD or in exercise regulation. Therefore, these genes should be regarded as potential regulatory nodes rather than core mechanistic genes [85–88]. Thus, the results of the PPI network analysis are more suitable for screening potential regulatory nodes, rather than directly inferring their biological causal relationships. Subsequent studies will need to combine gene knockout or inhibition experiments to further validate the specific functions of these nodes in AD.

Figure 1Heatmaps depicting significantly differentially expressed genes in ABX‐ and methionine‐restriction groups versus controls. (A) Top 20 significantly DEGs from RNA‐seq of male APPPS1‐21 mice treated with antibiotics (ABX; *N* = 6) compared with male APPPS1‐21 controls (*N* = 6); data were obtained from the GEO database (accession: GSE185407). (B) Top twenty20 DEGs from RNA‐seq of male 5xFAD mice treated with antibiotics (ABX; *N* = 6) compared with male 5xFAD controls (*N* = 3); data were obtained from the GEO database (accession: GSE154428). (C) Top 20 DEGs from RNA‐seq of male AD mice subjected to a methionine‐restricted (MR) diet (*N* = 11) versus male AD mice fed standard chow (*N* = 11); data were obtained from the GEO database (accession: GSE164618). “con” denotes control; “exp” denotes antibiotic or dietary intervention. Differential expression analysis was performed using the DESeq2 and limma R packages. Genes were log2‐transformed, and DEGs were defined as those with absolute log2 fold change > 1 and a false discovery rate (FDR) < 0.05.

**Figure:**
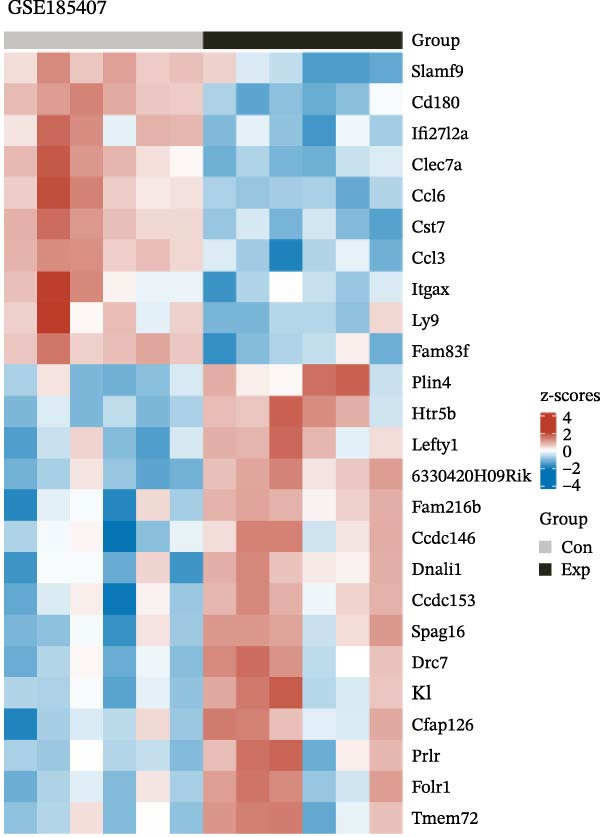
(A)

**Figure:**
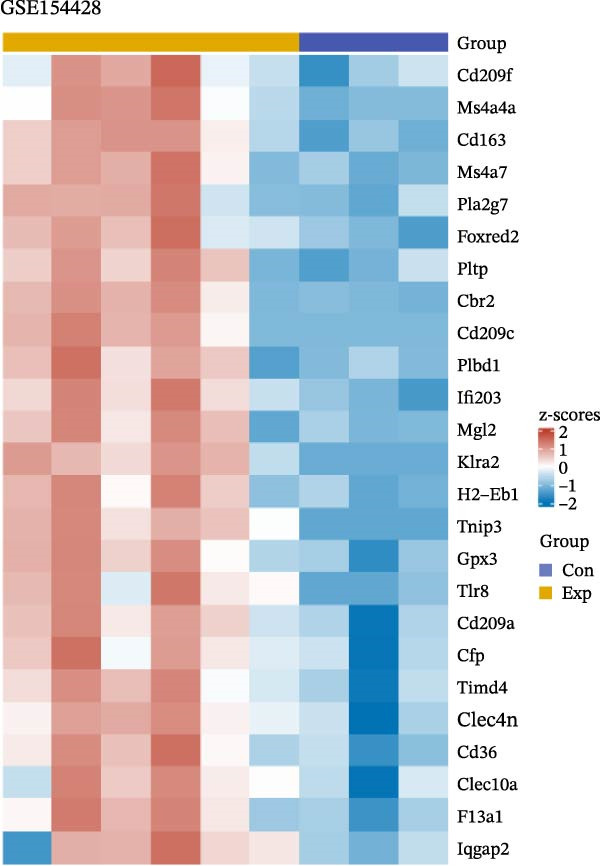
(B)

**Figure:**
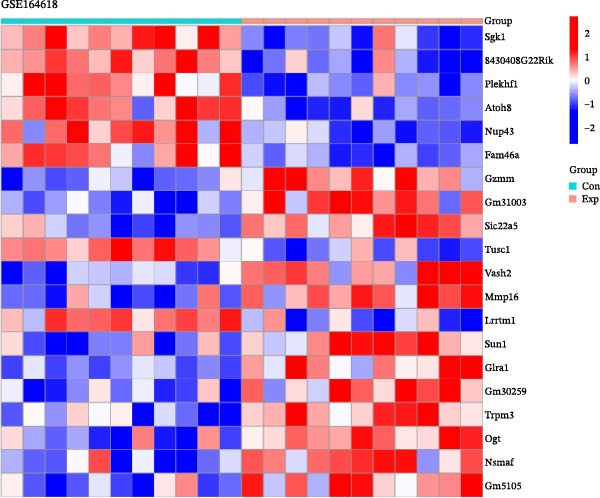
(C)

Figure 2Functional enrichment and protein–protein interaction (PPI) network analyses of DEGs in AD mouse models under ABX treatment and methionine restriction. (A) GO (left) and KEGG (right) enrichment analyses of RNA‐seq data from male APPPS1−21 mice treated with antibiotics versus APPPS1−21 controls; data were obtained from the GEO database (accession: GSE185407). (B) GO (left) and KEGG (right) enrichment analyses of RNA‐seq data from male 5xFAD mice treated with antibiotics versus 5xFAD controls; data were obtained from the GEO database (accession: GSE154428). (C) GO (left) and KEGG (right) enrichment analyses of RNA‐seq data from male AD mice on a methionine‐restricted diet versus males on standard chow; data were obtained from the GEO database (accession: GSE164618). For each dataset, GO and KEGG enrichment were conducted in R (v4.4.2) using the clusterProfiler, enrichplot, and ggplot2 packages; terms with both *p*‐value and *q*‐value <0.05 were considered significantly enriched. (D) A Venn diagram illustrating the intersection of all DEGs across the three datasets. (E) A protein–protein interaction (PPI) network constructed from the DEGs common to the three datasets (*n* = 9151). In the PPI network, nodes represent proteins and edges represent experimentally or computationally inferred interactions; highly connected nodes often indicate proteins that contribute substantially to network stability and function. The PPI network was built using the STRING database with an interaction confidence threshold >0.95, and subsequently visualized and reconstructed in Cytoscape (v3.6.1). (F) The top 20 genes ranked by node degree within the reconstructed PPI network.

**Figure:**
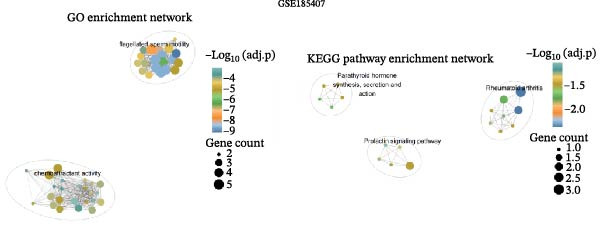
(A)

**Figure:**
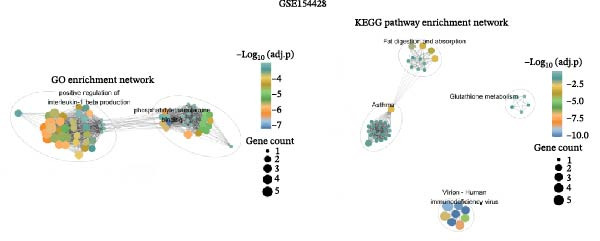
(B)

**Figure:**
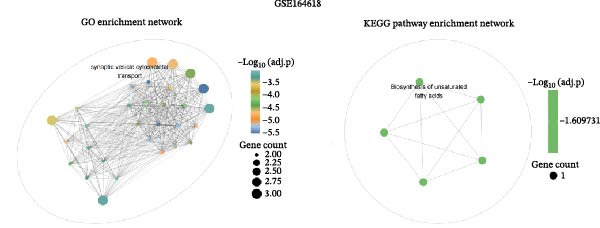
(C)

**Figure:**
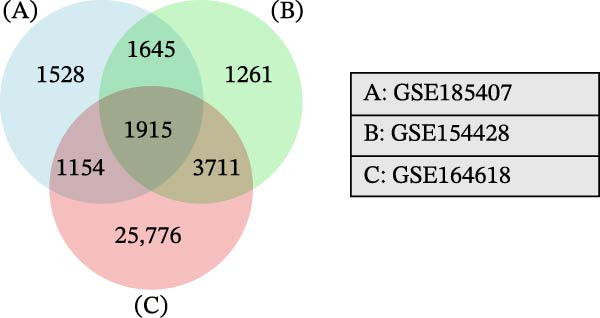
(D)

**Figure:**
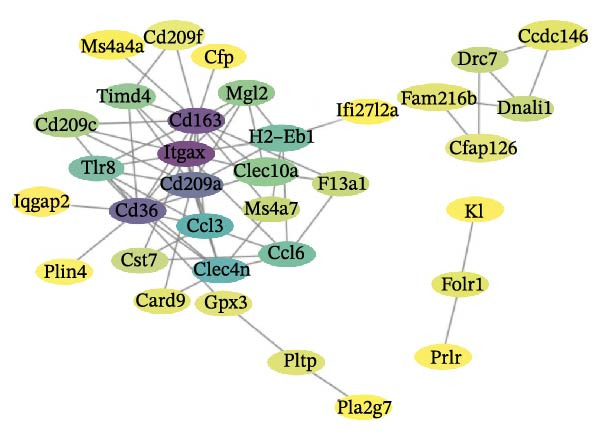
(E)

**Figure:**
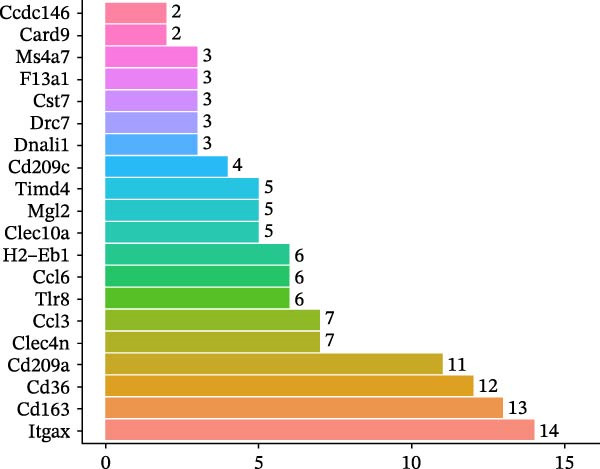
(F)

Systematic interrogation of these shared DEGs and hub proteins not only reveals convergent pathways through which disparate interventions modulate the gut–brain axis, but also furnishes molecular rationale for early therapeutic interception in AD. Notably, recent work suggests that exercise may confer cognitive protection via analogous gut microbiota–brain axis (GBA) mechanisms: regular physical activity remodels gut microbial composition, elevates short‐chain fatty acid (SCFA) levels, and modulates inflammatory cascades such as the Tlr4/NF‐κB axis, thereby mitigating neuroinflammation and enhancing neural plasticity [89]. Therefore, future investigations should prioritize elucidating how exercise influences AD‐related signaling networks via GBA‐mediated pathways to restore neuroimmune homeostasis and synaptic function. We further emphasize that multi‐omics integrative approaches—combining transcriptomics, metabolomics, and single‐cell omics—will be pivotal for deciphering the dynamic interplay between microbial metabolites (e.g., SCFAs) and central nervous system processes, and may open avenues for precision microbiota‐based interventions in AD.

### 5.2. Multilayered Mechanisms by which Exercise Remodels the Gut Microbiome

Exercise, as a non‐pharmacological intervention, reshapes the gut microbial ecosystem via multiple, interlocking mechanisms. First, on the immunoregulatory axis, moderate exercise enhances intestinal IgA secretion and modulates the Th17/Treg balance, thereby suppressing pro‐inflammatory responses and promoting release of anti‐inflammatory cytokines such as IL‐10, which together create a more permissive milieu for probiotic colonization [90]. Second, via neuroendocrine modulation, physical activity activates the hypothalamic–pituitary–adrenal (HPA) axis and alters secretion of enteric neuropeptides, optimizing intestinal motility rhythms and thereby reducing retention time of pathogenic taxa [91]. Metabolic reprograming is another key dimension: exercise‐induced skeletal muscle contraction increases concentrations of SCFAs in the cecum [92]; these microbial metabolites lower luminal pH to inhibit pathogen overgrowth and can cross the BBB to modulate microglial phenotype and attenuate neuroinflammation [93]. In addition, exercise favorably alters bile acid metabolism, supporting the expansion of beneficial genera such as Bifidobacterium [94]. Exercise also helps preserve gut barrier integrity and, through modulation of intestinal permeability, influences immune system maturation and surveillance [95]. Such exercise‐induced gut adaptations may selectively favor microbes capable of thriving under the altered physiological conditions, thereby reshaping community composition [96]. Although these studies collectively illuminate potential routes by which exercise modulates the microbiome, further work is required to delineate the precise, species‐specific mechanisms by which physical activity alters particular bacterial taxa.

### 5.3. Potential Mechanisms and Translational Prospects of Exercise–Microbiota Interactions in Ameliorating AD

Recent reviews have highlighted that exercise can regulate the gut microbiota and its metabolites, thereby influencing the occurrence and progression of AD through the gut–brain axis. Leng et al. [32] emphasized in their review that exercise significantly improves the diversity and composition of the gut microbiota, alters the metabolism of key neurotransmitters (such as glutamate and aspartate), and alleviates neuroinflammation and maintains BBB integrity by enhancing the production of metabolites like SCFAs. Similarly, Qin et al. [97] pointed out that regular exercise increases the abundance of beneficial bacteria such as *Akkermansia muciniphila*, enhances the expression of BDNF, reduces gut permeability, and prevents inflammatory factors like LPS from entering the bloodstream. These reviews collectively suggest that exercise exerts neuroprotective effects through the “exercise–microbiota–brain axis” and further validate the potential of gut microbiota interventions in AD treatment.

Symbiotic experiments have confirmed that, following the establishment of shared blood circulation between AD model mice and wild‐type mice, Aβ deposition in the brain significantly decreased [98], suggesting that peripheral circulating factors and gut‐derived metabolites may regulate central pathology through multiple pathways. Recent studies have gradually unveiled that exercise may participate in this cross‐organ regulatory process through the GBA. However, it should be emphasized that this field is still in a rapidly developing stage, and some mechanistic evidence remains speculative. Existing research suggests that exercise may improve AD pathology through the following pathways: (1) SCFAs–microglia axis: Exercise can increase the abundance of butyrate‐producing bacteria (e.g., Lachnospiraceae), elevate SCFA levels, and promote the transformation of microglia from the pro‐inflammatory M1 phenotype to the neuroprotective M2 phenotype via class HDAC inhibition mechanisms [99]; (2) Tryptophan metabolism pathway remodeling: Exercise increases the metabolic capacity of Rothia species for tryptophan, reduces KYN pathway activity, and enhances serotonin levels, thereby decreasing the accumulation of neurotoxic metabolites and improving synaptic plasticity [100]. These mechanisms have been cross‐supported in various AD mouse models.

However, current evidence regarding “gut‐derived exosomal miRNA (e.g., miR‐21a‐5p) crossing the BBB to regulate BACE1 and reduce Aβ generation” mainly comes from in vitro experiments or non‐AD disease models [101], and lacks direct validation of its causal effects in the AD context. Therefore, such mechanisms should be regarded as speculative hypotheses awaiting verification rather than established translational pathways. Moreover, consistent microbiota changes across studies remain limited. Trends have shown that AD patients and AD models typically exhibit an increase in Bacteroidetes and a decrease in Firmicutes, while exercise interventions often increase beneficial bacteria such as Akkermansia, Lactobacillus, and Bifidobacterium [89]. However, these results are sensitive to experimental conditions, such as sequencing technology (16S rRNA vs., shotgun metagenomics), host genetic background, gender, age, and exercise intensity [102]; the latest human meta‐analysis also indicated that AD‐related microbiota features exhibit high heterogeneity, with weak consistency across populations.

Thus, the exercise–microbiota–brain axis model proposed in this study is better understood as a trend‐based framework integrating evidence from multiple studies, rather than a fully established causal chain. In terms of translational potential, exercise, as a safe, low‐cost, and multi‐target intervention, offers an attractive new direction for AD treatment. However, its clinical translation still requires higher‐quality evidence, including stratified analysis of populations with different microbiota characteristics, longitudinal monitoring of SCFAs and peripheral inflammation markers, and clinical trials evaluating in conjunction with plasma p‐tau, NfL, and other biomarkers. Future systematic studies combining multi‐omics integration (transcriptomics, metabolomics, single‐cell sequencing) and metagenomics will help uncover key nodes in exercise regulation of the GBA and lay a more solid theoretical foundation for precision intervention strategies in AD.

## 6. Discussion and Perspectives

### 6.1. Current Limitations of the Research

Although exercise has exhibited pleiotropic protective effects in AD models in recent years, the mechanistic causal chain remains incompletely delineated. Overall, while physical activity shows potential cognitive benefits, outcomes across studies are inconsistent. A 2022 systematic review and meta‐analysis reported that exercise interventions produced significant improvements in cognitive scores among AD patients. However, not all trials found positive effects: Frederiksen et al. [103] observed in a randomized controlled trial of patients with mild AD that 16 weeks of moderate‐to‐high‐intensity aerobic exercise did not significantly change serum neurofilament light chain (NfL) levels. Likewise, Sewell et al. [104] reported in a 6‐month bicycle exercise RCT among cognitively normal older adults that plasma Aβ42/40, p‐tau181, GFAP, and NfL levels did not differ pre‐ versus post‐intervention. These findings indicate that standalone exercise interventions may have limited short‐term effects on circulating AD biomarkers. By contrast, a cross‐sectional study found that higher levels of physical activity were associated with lower plasma p‐tau217 and NfL and with better cognitive performance, suggesting that exercise’s benefits may relate to modulation of neuronal injury markers [105].

Most extant studies are correlational, and true “causal‐intervention” investigations leveraging genetic or pharmacological perturbations remain scarce. Preliminary causal evidence does exist for specific pathways: blockade of BDNF/TrkB signaling markedly attenuates exercise‐induced enhancements in synaptic plasticity [106, 107]; inhibition of SIRT1 abrogates exercise‐driven mitochondrial reprograming and metabolic adaptations [108]. Moreover, Tlr4 knockout models display resistance to gut‐derived inflammatory drivers of central neuroinflammation [109, 110], and convergent data indicate that exercise can downregulate the TLR4/NF‐κB cascade [111], positioning this axis as a putative hub for exercise‐mediated gut–brain modulation. Nonetheless, the mechanistic literature is limited by several recurring shortcomings: (1) a paucity of conditional knockout models and pathway‐specific blockade experiments, such that causal chains frequently rely on inference; (2) an absence in many studies of control groups exposed to environmental enrichment without exercise, making it difficult to ascribe effects specifically to physical activity; and (3) substantial heterogeneity in responses across different AD transgenic models, which underscores the model‐dependance of exercise effects and necessitates careful model‐specific interpretation when synthesizing preclinical evidence. For example, in APP/PS1 models—where Aβ pathology predominates—voluntary wheel running or treadmill training consistently reduces Aβ plaque burden and soluble Aβ40/42 levels and improves synaptic function and proteostasis, implying that exercise in this model chiefly facilitates Aβ clearance and metabolic regulation [112–115]. By contrast, in the 3xTg‐AD model, which harbors both Aβ and tau pathology, exercise outcomes are notably inconsistent: some studies report improvements in Aβ burden, neuroinflammation, and mitochondrial function, whereas others fail to demonstrate significant effects on tau hyperphosphorylation or Aβ levels, reflecting the greater mechanistic complexity in mixed‐pathology contexts [38, 116–118]. In the rapidly progressive 5xFAD model, more specific pathways have recently been delineated: exercise activates hippocampal PPARα and upregulates ADAM10, promoting non‐amyloidogenic APP processing and thereby reducing Aβ production and deposition [20, 119, 120]. (4) Sex is an underappreciated but potentially decisive biological variable in AD exercise research. Despite higher clinical incidence of AD in women, many preclinical studies—particularly those using APP/PS1 and 5xFAD models—preferentially or exclusively employ male animals to avoid estrous‐cycle–related variation, producing results whose generalizability to females is uncertain [121]. Only a minority of studies include female subjects or perform sex‐stratified analyses; however, those that do often reveal sexually dimorphic responses. For instance, a 2025 APP/PS1 study of long‐term resistance training documented clear sex‐dependent effects: females exhibited improvements in short‐term memory, reduced hyperactivity, increased VEGF, and decreased Aβ plaque burden after intervention, whereas males predominantly showed enhancements in learning and long‐term memory, elevated IGF‐1 and BDNF, and behavioral gains without Aβ reduction [122]. Similar sex‐dependent neuroprotection favoring females has been reported in TgF344‐AD rats, further underscoring sex as a critical determinant of exercise efficacy [123]. Although some clinical trials have begun to stratify randomization by sex, systematic reporting and analysis of sex differences remain limited, substantially impeding effective translation from preclinical models to heterogeneous human populations [124, 125]. This study acknowledges the issues of model heterogeneity and gender imbalance, and notes that these factors may have a potential impact on the generalizability of the results. Therefore, future research should fully consider gender differences during the design phase, include female animals, and conduct independent gender analyses to accurately reveal the true benefits of exercise interventions and gender‐specific mechanisms. This approach will help avoid over‐interpretation of the results and enhance the comprehensiveness and reliability of the research.

Despite a substantial body of supportive literature, both preclinical and clinical evidence include non‐significant and discordant findings, indicating that exercise effects are neither universal nor uniform but are influenced by disease stage, exercise modality, and interindividual biological variability [115, 117]. In animal studies, several experiments in complex‐pathology models such as 3xTg‐AD have reported that long‐term voluntary exercise fails to significantly ameliorate Aβ or global pathology, and some experiments document a dissociation between pathological improvement and behavioral outcomes, wherein reduced Aβ burden does not translate into measurable cognitive benefit [117, 126]. Moreover, high‐intensity forced exercise can activate the HPA axis and induce chronic stress responses that may negate or reverse exercise’s neuroprotective effects [127–129]. Clinically, a randomized trial of 200 patients with mild AD found that moderate‐to‐high‐intensity aerobic exercise did not outperform control on primary cognitive endpoints and produced benefits primarily in neuropsychiatric symptoms; other trials have been underpowered to detect between‐group differences owing to small sample sizes and limited statistical power [126, 130, 131]. Meta‐analyses employing funnel plots and Egger’s tests have also detected potential publication bias, suggesting that negative studies may be underreported and that the benefits of exercise could be overestimated if null results are not fully represented in the literature [132, 133]. Taken together, these limitations argue for cautious interpretation of existing data and for rigorous, well‐powered, mechanistically informed trials to determine the conditions under which exercise delivers reproducible benefit in AD.

### 6.2. Key Challenges to Implementation Feasibility

Although the exercise–microbiota axis demonstrates clear, multi‐target benefits in animal models, its real‐world, and clinical implementation faces multiple hurdles. Cognitive decline, motivational deficits and executive dysfunction in AD patients substantially impede long‐term adherence to structured exercise regimens, making compliance the primary constraint on intervention efficacy [134, 135]. Improving adherence will require multifaceted strategies, including individualized exercise prescriptions, incorporation of gamified and cognitive‐stimulating elements (e.g., VR‐based training), caregiver‐supported supervision, wearable‐device–mediated real‐time feedback, and robust social‐support frameworks [136–138].

Moreover, whereas animal studies using fecal microbiota transplantation (FMT) have functionally linked exercise‐induced microbial shifts to phenotypic outcomes [139], high‐quality human trials verifying that exercise benefits are mediated by the microbiome remain scarce. Most clinical studies focus on cognitive and physical endpoints while treating microbiome composition, metabolic pathways, and inflammatory markers as secondary or exploratory outcomes [140], leaving the cross‐species translational evidence chain incomplete. Crucially, clinical effects of exercise exhibit substantial inter‐individual heterogeneity: sex, age, baseline fitness, APOE ε4 genotype, and baseline gut microbiome configuration can all modulate both the magnitude and direction of response to exercise [141]. These factors collectively constitute “individualized noise” that impedes the development of precision exercise prescriptions and represent a major bottleneck for clinical scalability. In sum, to achieve sustainable and generalizable benefit in real‐world settings, exercise interventions must overcome barriers of poor adherence, limited mechanistic validation in humans, and pronounced inter‐individual variability.

### 6.3. Prospects for Multimodal Integrative Interventions and Research Priorities

Given that AD pathology reflects multisystem dysregulation—including metabolic perturbation, chronic inflammation, microbial dysbiosis, and synaptic degeneration—monotherapies are unlikely to produce durable, substantial clinical effects. Consequently, multimodal integrative interventions are emerging as a principal research direction. Randomized trials such as COCOA and PONDER suggest that combining exercise with dietary modification, cognitive training, or targeted nutritional supplementation yields synergistic effects across metabolic, inflammatory, and neurofunctional domains, and may produce more robust cognitive benefits than single‐modality approaches [142–146].

To accelerate precision and clinical translation of exercise‐based strategies in AD, future work should prioritize: (1) mechanism‐integrated clinical trials that concurrently evaluate gut microbiota composition, metabolomic signatures, peripheral fluid biomarkers (e.g., p‐tau, NfL), neuroimaging endpoints and cognitive outcomes to build cross‐dimensional validation frameworks; (2) standardized pathways for multimodal interventions, defining optimal combinations and dosing of exercise, dietary regimens (e.g., Mediterranean diet, prebiotics), cognitive training, and metabolic pharmacotherapies; (3) individualized prescriptions driven by omics and digital technologies—leveraging genotype (e.g., APOE ε4), microbiome phenotyping, wearable‐derived behavioral metrics and AI‐based predictive models to tailor interventions; and (4) translational platforms that integrate nonhuman primate models and organoid systems to validate key mechanisms and improve the reliability of animal‐to‐human extrapolation. These avenues will underpin the development of precise, implementable, and scalable exercise‐based interventions for AD.

## 7. Conclusion

This review systematically synthesizes mechanistic evidence from rodent models of AD to highlight the multitarget therapeutic potential and translational value of exercise interventions. Accumulating data indicate that treadmill training and swimming ameliorate Aβ deposition, Tau hyperphosphorylation, mitochondrial dysfunction, neuroinflammation, and synaptic degeneration through modulation of key signaling pathways, including BDNF/TrkB, SIRT1/PGC‐1α, and Nrf2/ARE. The gut–brain axis likely plays a pivotal role in exercise‐induced neuroprotection by reshaping gut microbiota composition and metabolites, thereby influencing neuroimmune interactions and cognitive function. Regulatory nodes identified through transcriptomic and molecular network analyses may serve as critical bridges between peripheral and central signaling. Despite significant progress, multiple challenges remain, including mechanism validation, optimization of intervention parameters, and translation to heterogeneous populations. Future research should focus on using conditional gene models, specific pathway inhibition, and nonexercise control groups to further elucidate the specific mechanisms of exercise interventions. Additionally, combining multimodal interventions (exercise, nutrition, gut microbiota modulation) with biomarker‐driven clinical validation will contribute to the advancement and verification of personalized intervention strategies for AD. This research direction should focus on conceptual theoretical progress and future validation, rather than the current readiness for clinical application.

**Nomenclature**
3xTg‐AD: Triple‐transgenic Alzheimer’s disease (mouse model)
5xFAD: Transgenic mouse model carrying five familial AD mutations
Aβ: Amyloid‐beta (β‐amyloid)
ABX: Antibiotics (antibiotic treatment)
AD: Alzheimer’s disease
ADAM10: A disintegrin and metalloproteinase 10
AI: Artificial intelligence
AKT: Protein kinase B (Akt)
APOE: Apolipoprotein E
APP: Amyloid precursor protein
APP/PS1: Double‐transgenic AD mouse model (amyloid precursor protein/presenilin 1)
ARE: Antioxidant response element
BACE1: β‐site APP‐cleaving enzyme 1 (beta‐secretase)
BCAA: Branched‐chain amino acid
BBB: Blood–brain barrier
BDNF: Brain‐derived neurotrophic factor
BMT: Barnes maze (test)
BPSD: Behavioral and psychological symptoms of dementia
CDNF: Cerebral dopamine neurotrophic factor
CNS: Central nervous system
COCOA: Coaching for Cognition in Alzheimer’s (multimodal intervention trial)
DEGs: Differentially expressed genes
EPM: Elevated plus maze (test)
ERK: Extracellular signal–regulated kinase
FMT: Fecal microbiota transplantation
GBA: Gut–brain axis
GDNF: Glial cell line–derived neurotrophic factor
GFAP: Glial fibrillary acidic protein
GO: Gene Ontology
GPx: Glutathione peroxidase
GSH: Glutathione (reduced form)
GST: Glutathione S‐transferase
HDAC: Histone deacetylase
HO‐1: Heme oxygenase‐1
HPA: Hypothalamic–pituitary–adrenal (as in HPA axis)
HSP70: Heat shock protein 70
IDO: Indoleamine 2,3‐dioxygenase
IgA: Immunoglobulin A
IL‐1β: Interleukin‐1β
IL‐6: Interleukin‐6
IL‐1: Interleukin‐10
JNK: c‐Jun N‐terminal kinase
KEGG: Kyoto Encyclopedia of Genes and Genomes
KYN: Kynurenine
LPS: Lipopolysaccharide
LRP1: Low‐density lipoprotein receptor‐related protein
1LTD: Long‐term depression
LTM: Long‐term memory
LTP: Long‐term potentiation
MAPK: Mitogen‐activated protein kinase
MDA: Malondialdehyde
miRNA: microRNA
MR: Methionine restriction (diet)
MWM: Morris water maze (test)
NF‐κB: Nuclear factor kappa B
NfL: Neurofilament light chain
NGF: Nerve growth factor
NMDA: N‐methyl‐D‐aspartate (as in NMDA receptor)
NO: Nitric oxide
NOR: Novel object recognition (test)
NQO1: NAD(P)Hquinone oxidoreductase 1
NRF1: Nuclear respiratory factor 1
Nrf2: Nuclear factor erythroid 2‐related factor 2
OFT: Open field test
p‐tau: Phosphorylated tau protein
PGC‐1 α: Peroxisome proliferator–activated receptor‐γ coactivator‐1α
PI3K: Phosphoinositide 3‐kinase
PICOS: Population, Intervention, Comparison, Outcomes, Study design
PKC: Protein kinase C
PONDER: Protein, Omega‐3 and Vitamin D Exercise Research (trial)
PPI: Protein–protein interaction
PRISMA: Preferred Reporting Items for Systematic Reviews and Meta‐Analyses
PSD‐95: Postsynaptic density protein 95
ROCK1: Rho‐associated protein kinase 1
ROS: Reactive oxygen species
SAM: S‐adenosylmethionine
SAMP8: Senescence‐accelerated mouse‐prone 8 (aging mouse model)
SCFA: Short‐chain fatty acid
SIRT1: Sirtuin 1 (silent information regulator 1)
SOD: Superoxide dismutase
STM: Short‐term memory
STZ: Streptozotocin (neurotoxin used to induce an AD model)
SYRCLE: Systematic Review Centre for Laboratory Animal Experimentation (risk‐of‐bias tool)
Tg2576: Transgenic AD mouse model (APP Swedish mutation)
TgCRND8: Transgenic AD mouse model (APP Swedish + Indiana mutations)
Th17: T helper 17 cell
TNF‐α: Tumor necrosis factor‐α
Treg: Regulatory T cell (T regulatory cell)
TrkB: Tropomyosin receptor kinase B
TRP: Tryptophan
TST: Tail suspension test
VEGF: Vascular endothelial growth factor
VR: Virtual reality
YM: Y‐maze (task/test for working memory).

## Author Contributions

All authors have made a substantial contribution to this article.

## Funding

This study was supported by the Fundamental Research Funds for the Central Universities (Grant No. 2025023).

## Conflicts of Interest

The authors declare no conflicts of interest.

## Supporting Information

Additional supporting information can be found online in the Supporting Information section.

## Supporting information


**Supporting Information** Table S1: Search strategy used for searched databases. Table S2: Evaluation results of SYRCLE animal experiment risk assessment tool. Figure S1: PRISMA flow diagram.

## Data Availability

The data that support the findings of this study are available from the corresponding author upon reasonable request.

## Appendix

All transcriptomic datasets reanalyzed in this study were obtained from the National Center for Biotechnology Information (NCBI) Gene Expression Omnibus (GEO) public repository (accession numbers: GSE185407, GSE154428, and GSE164618). These datasets are publicly accessible through GEO; original raw data and accompanying metadata can be retrieved from the GEO database. Bioinformatic workflows used in this study—including transcriptomic data preprocessing, differential expression analysis, GO/KEGG functional enrichment, and protein–protein interaction (STRING) network construction—were implemented in the R environment. To ensure reproducibility, all analysis scripts and key parameter settings have been compiled and provided in the Supporting Information. This study did not generate new primary animal experimental data. Data employed for the literature review were drawn from published, peer‐reviewed sources and are fully cited in the References. For additional information regarding the datasets, analysis code, or computational workflow, please contact the corresponding author.
