# Supplementary material for: Mechanistic Investigation of Exercise Interventions in Rodent Models of Alzheimer’s Disease and Prospects for Clinical Translation
Source: Neural Plast. 2026 Mar 17;2026:6718671. doi: 10.1155/np/6718671 (PMC13140407; doi:10.1155/np/6718671)
Supplement: Supplementary file 1 — Supporting Information Table S1: Search strategy used for searched databases. Table S2: Evaluation results of SYRCLE animal experiment risk assessment tool. Figure S1: PRISMA flow diagram. [file NP-2026-6718671-s001.docx]

**Supplementary Materials**

**Table 1 Search strategy used for searched databases**

| **Database** | **Search Strategy** |
| --- | --- |
| PubMed | (  Alzheimer Disease[Mesh] OR "Alzheimer’s disease"[tiab] OR "Alzheimer disease"[tiab]   OR "AD"[tiab] OR "cognitive impairment"[tiab] ) AND (  Mice[Mesh] OR Rats[Mesh] OR "rodent*"[tiab] OR "mouse model*"[tiab]   OR "APP/PS1"[tiab] OR "5xFAD"[tiab] OR "3xTg-AD"[tiab]   OR "Tg2576"[tiab] OR "TgCRND8"[tiab] OR "STZ-induced"[tiab] ) AND (  Exercise[Mesh] OR "Physical Exertion"[Mesh] OR Running[Mesh] OR Swimming[Mesh]   OR "exercise"[tiab] OR "physical activity"[tiab] OR "running"[tiab]   OR "treadmill"[tiab] OR "forced running"[tiab] OR "voluntary wheel running"[tiab]   OR "swimming"[tiab] ) AND (  Amyloid beta-Peptides[Mesh] OR Tau Proteins[Mesh] OR Synapses[Mesh]   OR Neuroinflammation[Mesh] OR Mitochondria[Mesh]  OR "amyloid beta"[tiab] OR "Aβ"[tiab] OR "tau phosphorylation"[tiab]  OR "synaptic plasticity"[tiab] OR "LTP"[tiab] OR "LTD"[tiab]  OR "neuroinflammation"[tiab] OR "microglia"[tiab]  OR "mitochondrial dysfunction"[tiab] ) |
| WOS | TS = (  ("Alzheimer* disease" OR "AD" OR "cognitive impairment")  AND  ("mouse" OR "mice" OR "rodent*" OR "APP/PS1" OR "5xFAD"   OR "3xTg-AD" OR "Tg2576" OR "TgCRND8" OR "STZ-induced")  AND  ("exercise" OR "physical activity" OR "running" OR "treadmill"   OR "voluntary wheel running" OR "forced running" OR "swimming")  AND  ("amyloid beta" OR "Aβ" OR "tau phosphorylation" OR "synaptic plasticity"   OR "LTP" OR "LTD" OR "neuroinflammation" OR "mitochondria") ) |

**Identification of studies via databases and registers**

Records removed *before screening*:

Duplicate records removed (n = 158 )

Records identified from:

Pubmed (n = 667)

Web of Science (n = 689)

**Identification**

Records screened

(n = 1198 )

Records excluded

(n = 1089 )

Reports sought for retrieval

(n = 109 )

Reports not retrieved

(n = 3 )

**Screening**

Reports assessed for eligibility

(n = 106 )

Reports excluded:

Studies not involving exercise interventions (n = 39 )

Non-AD animal models (n = 36 )

Lack of mechanistic outcomes (n = 16 )

Studies included in review

(n = 15 )

**Included**

Figure 1. PRISMA flow diagram.

**Table 2 Evaluation results of SYRCLE animal experiment risk assessment tool**

| **Intervention Type** | **Included Study** | **Selection Bias** | | | **Performance Bias** | | **Detection Bias** | | **Attrition Bias** | **Reporting Bias** | **Other Bias** | **Total Score** |
| --- | --- | --- | --- | --- | --- | --- | --- | --- | --- | --- | --- | --- |
|  |  | **Sequence Generation** | **Baseline Characteristics** | **Allocation Concealment** | **Random Housing** | **Blinding** | **Random Outcome Assessment** | **Blinding** | **Incomplete Outcome Data** | **Selective Outcome Reporting** | **Other sources of bias** |  |
| Running | Moore KM 2016 | 2 | 2 | 1 | 1 | 1 | 1 | 1 | 2 | 2 | 2 | 15 |
| Running | Koo JH 2017 | 1 | 1 | 1 | 1 | 1 | 1 | 1 | 2 | 2 | 2 | 13 |
| Running | Zhang J 2018 | 1 | 1 | 1 | 1 | 1 | 1 | 1 | 1 | 2 | 2 | 12 |
| Running | Kim D 2019 | 1 | 1 | 1 | 1 | 1 | 1 | 1 | 2 | 2 | 2 | 13 |
| Running | Sun LN 2018 | 1 | 1 | 1 | 1 | 1 | 1 | 1 | 1 | 2 | 2 | 12 |
| Running | Haskins M 2016 | 1 | 1 | 1 | 1 | 1 | 1 | 1 | 1 | 2 | 2 | 12 |
| Running | Yuede CM 2009 | 1 | 1 | 1 | 1 | 1 | 1 | 1 | 1 | 2 | 2 | 12 |
| Running | Jiang L 2018 | 2 | 2 | 1 | 1 | 1 | 1 | 1 | 2 | 2 | 2 | 15 |
| Running | Lin TW 2015 | 1 | 1 | 1 | 1 | 1 | 1 | 1 | 1 | 2 | 2 | 12 |
| Swimming | Wu C 2018 | 2 | 1 | 1 | 1 | 1 | 1 | 1 | 1 | 2 | 2 | 13 |
| Swimming | Belviranl M 2019 | 1 | 1 | 1 | 1 | 1 | 1 | 1 | 1 | 2 | 2 | 12 |
| Swimming | Souza LC 2013 | 1 | 1 | 1 | 1 | 1 | 1 | 1 | 1 | 2 | 2 | 12 |
| Swimming | Widjaya MA 2023 | 1 | 1 | 1 | 1 | 1 | 1 | 1 | 1 | 2 | 2 | 12 |
| Swimming | Medhat E 2020 | 1 | 1 | 1 | 1 | 1 | 1 | 1 | 1 | 2 | 2 | 12 |
| Swimming | Souza LC 2017 | 1 | 1 | 1 | 1 | 1 | 1 | 1 | 1 | 2 | 2 | 12 |

Note: 2: Low risk; 1:Uncertain risk; 0: High risk.
